# Developing and optimizing a biocompatible tauopathy model using extracellular vesicle-mediated gene delivery

**DOI:** 10.3389/fmed.2025.1672046

**Published:** 2025-10-22

**Authors:** Samaneh Ghadami, Kristen Dellinger

**Affiliations:** Department of Nanoengineering, Joint School of Nanoscience and Nanoengineering, North Carolina A&T State University, Greensboro, NC, United States

**Keywords:** transfection, gene delivery, electroporation, exosomes, EGFP, extracellular vesicles, tauopathy, response surface methodology

## Abstract

**Introduction:**

Tauopathy models are essential in vitro systems for investigating tau-targeted therapies and advancing Alzheimer’s disease research. Extracellular vesicles (EVs), owing to their high biocompatibility, low toxicity, and reduced immunogenicity, represent promising carriers for gene delivery and disease modeling.

**Methods:**

We investigated the potential of EVs as a delivery system for the human four-repeat tau isoform lacking N-terminal sequences (4R0N) and enhanced green fluorescent protein (EGFP) into Neuro-2a cells. EV-mediated transfection efficiency was compared with conventional methods, including lentiviral and chemical (lipofectamine and polyethyleneimine, PEI) approaches. Response surface methodology (RSM) was used to optimize EV-mediated delivery parameters.

**Results:**

EVs successfully delivered large plasmid DNA into Neuro-2a cells, resulting in detectable tau and EGFP expression. Optimization via RSM further improved gene delivery efficiency and reproducibility compared to unoptimized EV preparations and conventional transfection methods.

**Discussion:**

These findings demonstrate that EVs can serve as a robust and biocompatible platform for tau gene delivery, providing a promising alternative to traditional transfection strategies for generating physiologically relevant tauopathy models.

## 1 Introduction

Alzheimer’s disease is the sixth-leading cause of death in the United States and the fifth-leading cause of death among aged Americans (1). Dementia, cognitive impairment, and memory loss are the common features of AD (2). Although AD is multifactorial in nature, the accumulation of amyloid-beta (Aβ) peptides and tau protein in the nervous system has been strongly implicated in driving the progression of its symptoms, including cognitive decline and neuronal dysfunction (3, 4). One approach to studying and simulating the cellular changes associated with AD is the use of *in vitro* tauopathy models, which provide valuable tools for investigating tau-related pathology and its role in disease progression (5–8). For example, changing the normal levels of tau protein through transfection methods has been shown to effectively mimic key aspects of tau dysfunction observed in AD (9). Transfection is a widely used method for genetically modifying cells for various purposes, including gene therapy (10–14), cellular metabolic studies (15), and individualized drug treatments (16). Different transfection methods are available, depending on the downstream application, cell type, and cell volume. However, each method has its advantages and limitations (14, 17). Transfection techniques can be categorized into physical, chemical, and biological methods (14).

Physical transfection methods include microinjection (18), optical transfection (19), particle bombardment using a gene gun (20), electroporation (21, 22), sonoporation (23), and magnetofection (24). While these methods avoid the side effects associated with other transfection techniques, they have limitations, such as reduced efficiency in delivering large genetic material and challenges in crossing the cell membrane. Additionally, these methods can be costly and may damage target cells (25).

Chemical transfection systems, including calcium phosphates, lipids (Lipofectamine), and cationic polymers (e.g., PEI), have gained attention because they can efficiently interact with DNA or RNA molecules. However, further research is needed to minimize their toxicity to target cells (25). For example, Lipofectamine has been extensively used to transfect Neuro-2a cells in neuronal studies (26–35).

Biological transfection such as viral transduction systems commonly used for transduction include adenoviruses, lentiviruses, and the Semliki Forest virus (14). Among these, lentiviral transduction offers long-lasting, though gradual, changes in the expression levels of target cells (14). Lentiviral vectors can accommodate up to 9 kbp of heterologous DNA and offer advantages such as low mutagenesis risk, low oncogenicity, and efficient integration into the host genome. These vectors are also not associated with pre-existing human immunity or human pathogenicity, making them effective for various cell types, including dividing cells, non-dividing cells, and those challenging to transduce, such as hematopoietic precursors, neurons, lymphoid cells, and macrophages (36). Lentiviral transduction can persist for several months, its efficiency and longevity make it an ideal choice for long-term studies (14). However, Virus-based gene vectors face batch-to-batch variability and challenges in scaling up production due to difficulties in developing stable packaging cell lines. Additional limitations include achieving high-titer, pure lentiviral particles and addressing safety concerns like insertional mutagenesis.

Extracellular vesicles (EVs) are nanosized particles encased in bilayers, containing a diverse range of lipid molecules, and are released by various cell types (37). They are not only natural carriers between various cells but also can be used for drug delivery applications (38–41). Several studies have also identified that EVs can deliver DNA as cargo (42–45). For example, Kang et al. showed that the loading of plasmid DNA is dependent on both the DNA size and dose. Considering these parameters, they introduced EVs as unique gene-delivery vehicles (46). Contradictory studies exist; Kahlert et al. confirmed the presence of double-stranded DNA within exosomes exceeding 10 KB (43). However, some scientific efforts have been reported that utilize EVs to deliver exogenous DNA into EVs via electroporation, depending on the size of the DNA molecule, with linear DNA strands shorter than 1 kbp exhibiting greater association with EVs compared to larger linear DNAs and plasmid DNAs (47).

Additionally, larger microvesicles encapsulated more linear and plasmid DNA than smaller, exosome-like EVs. They could load foreign DNA into EVs, but without the functional gene expression in the host cells (47). Another study used EVs to deliver siRNA to the mouse brain, demonstrating successful therapeutic effects in AD models (48). A comparison by Kanada et al. demonstrated that while both exosomes and plasma-derived vesicles can transport mRNA to host cells, only mRNA or plasmid DNA delivered by plasma membrane vesicles remains functional in recipient cells. They reported that large-EVs, but not small-EVs, derived from Lipofectamine-transfected HEK293 cells could be endogenously loaded with plasmids up to 8567 bp (49). These DNA plasmids were successfully incorporated into EVs, resulting in a quantifiable level of gene expression that was comparable to, and in some cases exceeded, the efficiency of conventional transfection methods (49). Focusing more on gene delivery studies to overcome the mentioned challenges, several studies affirm the efficacy of employing EVs for cell reprogramming purposes. Ortega-Pineda et al. demonstrated the use of EVs as a natural tool to transfect mouse embryonic fibroblasts, facilitating their transformation into neurons. They achieved this by loading Ascl1, Brn2, and Myt1l plasmids into exosomes using bulk electroporation, showcasing promising outcomes in cellular reprogramming (50). Another study by Rincon-Benavides et al. confirmed the proficient application of electroporation techniques with viral vectors in converting pluripotent stem cells into endothelial cells. This transformation was facilitated by equipping EVs with essential factors necessary for the conversion of stem cells (51). Moreover, Ortega-Pineda et al. illustrated the electro-transformation of pancreatic ductal epithelial cells into hormone-expressing cells using plasmid-loaded EVs. This approach enabled the expression of β cell markers in the ductal cells (52). In a separate investigation, Duarte-Sanmiguel et al. loaded miR-146a and Glut1 plasmids into EVs using electroporation. This study aimed to induce anti-tumor responses in myeloid-derived suppressor cells within a murine model of breast cancer (53). All these studies highlight the growing interest in novel bio-nature EV-based transfection methods for gene editing purposes, which is valuable due to their capabilities to evade the immune system and overcome the blood-brain barrier, a significant challenge in neurological diseases.

The loading of DNA into EVs can be achieved through incubation (54), electroporation (55), extrusion (55, 56) or freeze-thaw techniques (57). Due to the sensitivity of EVs there are many limitations to these techniques. For example, transfection reagents may remain in the sample and be a source of contamination, or many of the methods don’t have enough DNA loading efficiency or damage the EV integrity. Among all mentioned methods, electroporation is the best option to load EVs with nucleic acids, although this method has some disadvantages, such as aggregation or protocol variability (58). As a result, there is a need to standardize the approaches and characterize the loading efficiency and transfection efficacy for large plasmid DNA.

Response surface methodology (RSM) is a statistical optimization technique that efficiently studies the effects of multiple parameters and their interactions (59). By analyzing experimental data, RSM helps identify relationships between variables and their impact on outcomes. This technique is more time-efficient and advanced compared to traditional one-factor optimization methods, allowing for predictable responses and the assessment of model significance (60). Numerous optimization studies have benefited from the advantages of RSM. For example, RSM has been used to monitor the effect of cell density and expression time on the production level of reteplase enzyme (61), to evaluate the impact of polydopamine nanoparticles’ size, concentration, and laser fluence on FITC-dextran transfer to RAW264.7 mouse macrophage-like cells (62), and to optimize the transfer of plasmid psiRNA-hH1GFPzeo into HeLa cells using chitosan-albumin polymers as a delivery tool (63). These studies aimed to suggest optimal delivery conditions for efficient gene transfer.

To add to this growing depth of knowledge, this study investigates the capability of the EVs in transfection. EVs were extracted from Neuro-2a cells. Then, the desired DNA sequence (tau gene) was loaded in EVs using electroporation, and the host cells were exposed to these EVs to facilitate their tau-loaded EVs’ entrance in the Neuro-2a cells. Common transfection methods such as lentiviral vectors and chemical transfection tools such as lipofectamine or PEI were used as good sources to compare with.

**FIGURE 1:**
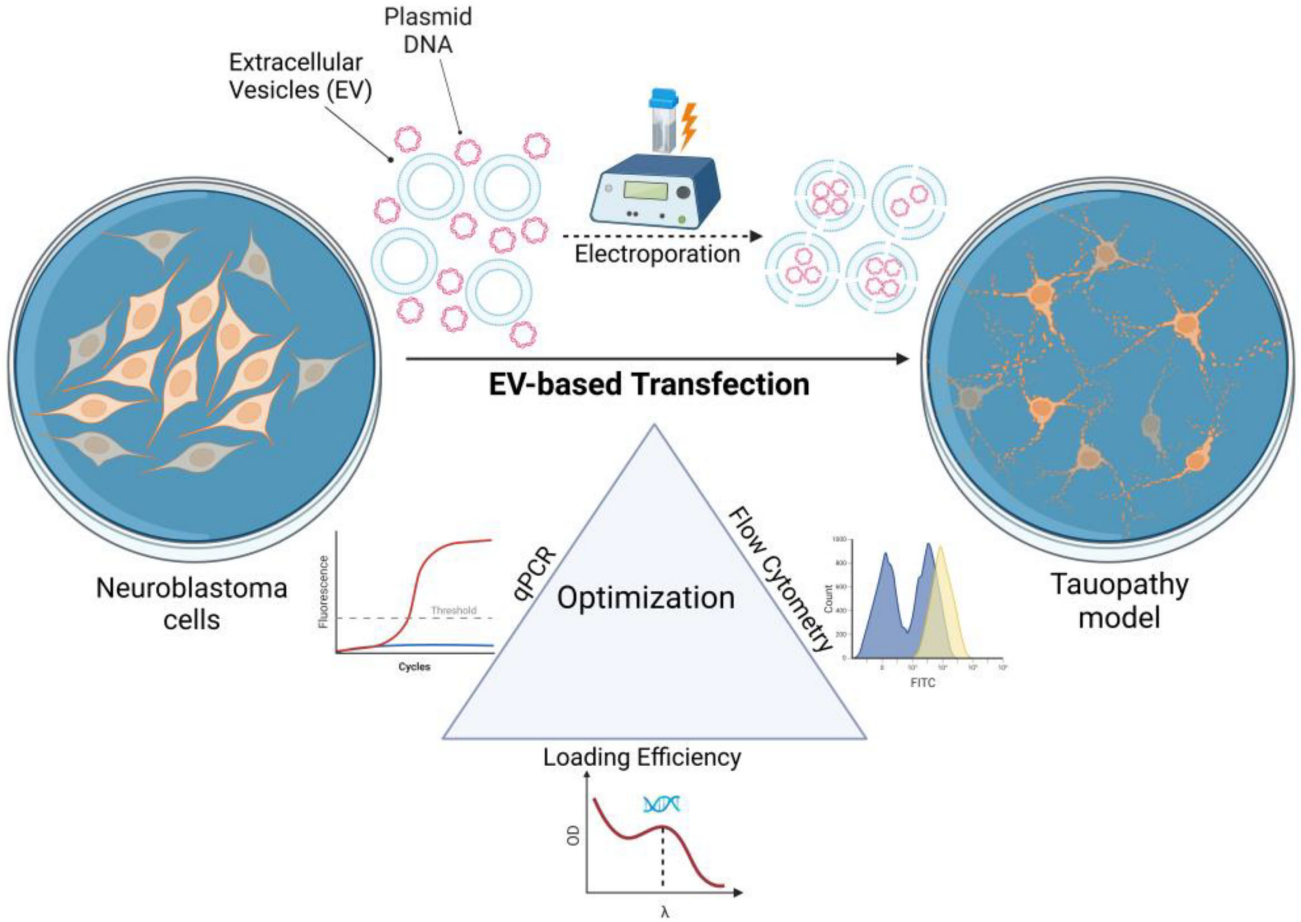
Schematic diagram illustrating the steps used in this study to optimize EV-based tau gene delivery in Neuro-2a cells using RSM. The optimization considered two variables [plasmid DNA amount (μg) and incubation time (h)] and three responses: qRT-PCR, flow cytometry analysis, and DNA loading efficiency (Created with BioRender.com).

Since transferring tau gene in Neuro-2a cells needs optimization to find the optimum DNA amount and incubation time, the Central Composite Design (CCD) method was applied to optimize the process and study the two-way interaction between variables. RSM, based on CCD, was employed using Design expert software 7.1.5 (State-Ease, Inc., USA, Windows operating system) to determine the experimental confirmation based on two variables (tau plasmid DNA amount and incubation time) and three responses (fluorescent intensity to measure the tau expression level, quantitative real-time polymerase chain reaction (qRT-PCR) analysis to evaluate the EV-associated DNA transfer to Neuro-2a cells and quantification of loaded DNA in EVs (Figure 1). The optimal conditions for significant EV-mediated delivery of the tau gene were then assessed in Neuro-2a cells. To the best of the authors’ knowledge, no previous research has addressed the optimization of EV-based transfection or fully characterized the combined effects of plasmid DNA concentration (μg) and incubation time (h) on gene transfer efficiency in Neuro-2a cells.

## 2 Materials and methods

### 2.1 Cell culture, plasmid preparation, and lentiviral vector production

HEK293 cells (gift from Dr. Christopher L. Kepley) were grown in complete Dulbecco’s Modified Eagle Medium, DMEM GlutaMAX (Thermo Fischer, 10569010) with 10% fetal bovine serum (FBS) and 1% antibiotics (penicillin and streptomycin). Neuro-2a cells were grown in complete Eagle’s minimum essential medium (EMEM) with 1% L-glutamine, 10% fetal bovine serum (FBS), and 1% antibiotics (penicillin and streptomycin). For cell detachment, Trypsin-EDTA (0.25%), phenol red (Thermo Fisher, 25200056), and sterile phosphate-buffered saline (PBS; Fisher Scientific, BP2944100) were used.

The lentiviral transduction process involved four plasmids: two third-generation core packaging plasmids (pMDLg/pRRE, Addgene 12251 and pRSV-Rev, Addgene 12253), an envelope-expressing plasmid (Addgene 12259), and the transfer plasmid pLJM1 EGFP-Tau plasmid [human four-repeat tau lacking the N-terminal sequences (4R0N) containing exons 1, 4 and 5, 7, and 9–13, intron 13, and exon 14, Addgene 108868]. Additionally, the control plasmid (pRRLSIN.cPPT.PGK-GFP.WPRE) (Addgene, 12252) was used. *E. coli* bacteria containing each mentioned plasmid cultured in LB broth media containing (containing 100 μg/ml Ampicillin) separately. Reaching the optical density of the bacteria growth to 1, the plasmid DNA extracted from the bacteria using PureLink™ HiPure plasmid Midiprep kit (Invitrogen, K210004). The plasmid DNA concentration is measured using Thermo Scientific NanoDrop Lite spectrophotometer. The A_260_/A_280_ ratio for all plasmid DNA samples was in the standard range.

Polyethyleneimine, linear, M.W. 25,000, PEI (Thermo Fisher, 043896.01) and the Lipofectamine™ 3000 Transfection Reagent (Invitrogen, L3000015) were used for chemical transfection.

To produce, purify, and concentrate the lentiviral vectors, the methods described in Follenzi and Naldini (64) were employed with slight modifications. Briefly, HEK293 cells are co-transfected with plasmids encoding the lentiviral genome, envelope proteins, and packaging proteins using a reagent, such as PEI. The transfection mixture was added dropwise to the cells with minimal disturbance, avoiding excessive pipetting or swirling. After 18 h, the medium was replaced with DMEM GlutaMAX supplemented with 10% FBS and 1% antibiotics.

After 48–72 h of culture to allow for lentiviral production, the cell culture supernatant containing lentiviral vectors was collected and filtered through a 0.45 μm filter to remove cell debris. Following filtration, the filtered supernatant was concentrated using ultracentrifugation at 32,000 rpm at 4 °C. Finally, the pellet containing pure lentiviral vectors was resuspended in PBS containing 1% BSA and finally stored in −80 °C. until further use. Neuro-2a cells were treated with the transduction mixture containing lentiviral vectors and polybrene (Sigma, TR-1003).

### 2.2 Flow cytometry, western blotting and ELISA

The produced lentiviral vectors were quantified using two methods: flow cytometry to assess functional vector titers through EGFP expression and ELISA to determine viral vector concentration based on absorbance of the p24 antigen. Flow cytometry, utilizing EGFP, serves as a functional assay to measure vector titers by assessing the efficiency of gene transfer. In contrast, the p24 antigen ELISA is a non-functional method that quantifies viral vector concentration based on the absorbance of the p24 antigen, a structural protein present in the viral capsid.

The production of lentiviral nanoparticles was quantified using the One Wash™ Lentivirus Titer Kit, HIV-1 p24 ELISA kit (Origin, TR30038), and flow cytometry using CytoFLEX flow cytometer (Beckman Coulter). The levels of HIV-1 p24 antigen in serial dilutions of the lentiviral stock were measured using the p24 ELISA kit according to the manufacturer’s instructions. Briefly, the HIV-1 p24 antigen was captured by microplate wells coated with anti-HIV-1 p24 capture antibodies, followed by reaction with a biotinylated detection antibody. After washing, the bound antigen was quantified spectrophotometrically.

The transduction efficiency of the lentiviral particles was evaluated in Neuro-2a cells using flow cytometry described in Follenzi and Naldini (64). Briefly, Neuro-2a cells were treated with serial dilutions of the lentiviral stock (10^3^, 10^4^, 10^5^, 10^6^, 10^7^, and 10^8^) for 72 h at 37 °C in a 5% CO_2_ incubator and finally processed and analyzed by flow cytometry to determine the level of transduction. In addition, flow cytometry was applied to get the optimum time expression of tau in Neuro-2a cells. Thus, a lentiviral titration was performed to optimize the number of vectors needed for the transduction of Neuro-2a cells. To quantify tau expression in EVs, tau levels were measured using the Novus Biological human tau ELISA kit (Colorimetric) (Novus Biologicals, NBP2-62749) following the manufacturer’s instructions. Briefly, EVs were collected from Neuro-2a cells overexpressing tau via chemical transfection (using PEI or Lipofectamine) and from lentiviral transduced Neuro-2a cells. Tau concentration (pg/mL) was determined using a human tau standard curve prepared specifically for this ELISA kit.

Western blotting was carried out using the following antibodies: primary anti-tau antibody [5B10] (Abcam, ab278070) and secondary goat anti-mouse (Abcam, ab205719) as validated in (65). The reagents were also used including Novex Tris-Glycine Transfer Buffer (Invitrogen, LC3675), TRIS-glycine-SDS running buffer (ThermoFisher, J61006.K7) No-stain protein labeling Reagent (ThermoFisher, A44717). Neuro-2a cells were treated with lentiviral vectors overnight, and 72 h post-transduction, proteins were extracted from lysed cells. Samples were combined with RIPA lysis buffer (Sigma, 20-188) at a 1:1 ratio and incubated for 1 h at 4 °C. Following the incubation, the samples were centrifuged at 16,000 *g* for 15 min at 4 °C, and the resulting pellet was discarded. Next, Laemmli SDS sample buffer, non-reducing (ThermoFisher J63615.AC) was added to the supernatant. The samples were then incubated at 90 °C for 6 min to facilitate denaturation. Finally, the prepared samples were loaded in 10% Mini-PROTEAN TGX Stain-Free Protein Gels (BIO-RAD, 4568034) to run the protein gel electrophoresis, transferred onto a nitrocellulose membrane, and probed with anti-tau and β-actin (primary anti-beta actin antibody, Abcam ab8226) antibodies. Finally, the blot image was obtained using the ECL Prime Western blotting detection reagent [Sigma (Cytiva), GERPN2236]. The protein ladders, including Precision Plus Protein Standard (BIO-RAD, 1610385) and Prestained Protein Ladder (Abcam, 116027), were used to monitor the protein separation during SDS gel electrophoresis. The total protein concentrations from the cells and EV lysates were measured using Pierce Bradford Plus Protein Assay Kits (ThermoFisher, 23236). The cells for both flow cytometry and confocal microscopy were fixed using paraformaldehyde 4% Image-I fixative solutions (ThermoFisher, I28800).

### 2.3 Assessment of tau gene transfer

Quantitative real-time polymerase chain reaction gene expression quantifications were performed using Power SYBR™ Green RNA-to-CT™ 1-Step Kit (ThermoFisher, 4389986). All reactions were performed in 96-well plates using Applied Biosystems 7500 Fast Real-Time PCR instrument. Reactions with extracted total RNA using Purlink RNA Mini Kit (Invitrogen, 12183018A) as input were performed in a total volume of 20 μl, comprising 2X Power SYBR Green RT-PCR mix, 125X of RT enzyme mix, 200 nM of each primer [EGFP (FWD): GAA CCG CAT CGA GCT GAA, EGFP (REV): TGC TTG TCG GCC ATG ATA TAG, GAPDH (FWD): CTG GGT GGA GTG TCC TTT ATC and GAPDH (REV): GGT GAG ACA GAT TGT GAG GTA G]. The primers were designed PrimerQuest™ program and ordered by Integrated DNA Technologies (IDT, Coralville, Iowa, USA) to target the tau gene and the GAPDH gene in the sample. All qRT-PCR reactions were performed in triplicate and Ct values were averaged and the calculations were performed using ΔΔCt method and presented as an averaged folding change. Normalization was performed using GAPDH as a reference gene. Negative controls including non-template controls and non-RT controls were included. Total RNA is extracted using PureLink™ RNA Mini Kit (Invitrogen, 12183018A) 48 h after incubating the Neuro-2a cells with a transfection mixture and RNA’s purity is measured through the absorption ratio at 260–280 nm (A_260_/A_280_).

### 2.4 EV isolation and characterization

Extracellular vesicles were isolated from the cell culture media using the Total Exosome isolation Reagent (Invitrogen, 4478359). Nanoparticle tracking analysis (NTA) of EVs isolated from Neuro-2a cells was done using a NanoSight LM10 instrument. Scanning electron microscopy (SEM) (Zeiss Auriga field-emission scanning electron microscope) and dynamic light scattering (DLS) were conducted to validate the size distribution and zeta potential of EVs. Western blotting of EVs derived from lentiviral transduced Neuro-2a cells was carried out using the following antibodies: primary anti-tau [5B10] (Abcam, ab278070), secondary goat anti-mouse (Abcam, ab205719), primary anti-CD81 [EPR4244] (Abcam, ab109201) and secondary goat anti-rabbit antibodies (ThermoFisher, A16104).

### 2.5 EV-based tauopathy models

The EV-based tauopathy models for all 12 experiments with different DNA amounts (μg) and incubation times (h) were prepared based on the methods used by Lamichhane et al. (47). Briefly, Neuro-2a cells were seeded on each well on a 6-well plate culture dish, and to prepare the transfection mixture the DNA was loaded in EVs using electroporation. For this purpose, DNA in Opti-MEM as the electroporation buffer was mixed with 2 × 10^10^ particles/mL EVs, earlier extracted from Neuro-2a cells, followed by a 10 s vortex. Finally, the transfection mixture for both samples and controls underwent electroporation (Voltage: 400, Resistance: 125 Ω, Capacitance: 50 μF, Pulse number: 2). Electroporation was conducted in 2 mm BTX™ Electroporation Cuvettes Plus™ (Fisher scientific, BTX620) using the Exponential Decay Wave Electroporation System (ECM^®^630, BTX Harvard apparatus) in a GenePulser electroporator. Then, all samples were passed through Nanosep centrifugal devices equipped with Omega membranes (300 KDa MWCO, Pall OD300C33) to eliminate free DNA and buffer constituents.

Post-electroporation, tubes were treated with 1 mM of EDTA to inhibit DNA aggregation and incubated at 37 °C for 30 min and then stored at 4 °C overnight to allow EVs to regain their integrity by resealing the EV membranes and packaging the DNA. The unloaded plasmid DNA is degraded by the RNase-Free DNase enzyme (Thermo Scientific, EN0521) and removed during the washing steps. The DNase enzyme was inactivated by adding 20 mM EDTA. Finally, the transfection was performed by adding the appropriate transfection mixture to the wells containing Neuro-2a cells, and then the cells were incubated at 37 °C. The transfection mixture for the control contains only EVs. After incubation, the cells were washed with PBS and the complete EMEM containing (10% FBS and 1% Penicillin/streptomycin) was added to each well. After 48 h, the cell fixation and total RNA extraction were performed to evaluate the gene expression levels for all 12 experiments.

### 2.6 DNA quantification

The transfection mixture containing tau loaded EVs in the samples and only EVs in controls were incubated at 90 °C for 6 min and then used for DNA quantification. DNA amount loaded in EVs was measured after EVs electroporation using a Quant-it PicoGreen assay kit (Life Technologies, P7589), following the manufacturer’s protocol. Known quantities of lambda DNA standard were similarly labeled with an ultrasensitive fluorescent nucleic acid stain for quantitating double-stranded DNA in solution to establish a standard curve for quantification.

### 2.7 Optimization of tau gene delivery in Neuro-2a cells; experimental design

A central composite design (CCD) was applied to optimize EV-based transfection in Neuro-2a cells, using Design Expert Software (version 7.1.5; Stat-Ease, Inc., USA). The experimental setup, summarized in Supplementary Table 1, varied plasmid DNA amount (μg) and incubation time (h) across five levels including −α, −1, 0, +1, +α which are defined in Supplementary Table 2. *F*-value and *p*-value indices were used to evaluate the significance of these variables and the model’s robustness.

## 3 Results

### 3.1 Chemical and lentiviral-based tauopathy models

To transfect cells using EVs, we first investigated common transfection methods to compare their efficiency with the proposed transfection technique using electroporated EVs. Tau overexpression in HEK293 cells, followed by lentiviral vector production, was confirmed 48 h post-transfection using the Discovery Echo rotary fluorescent microscope (Supplementary Figures 1a–d), while no expression was detected in control samples, as expected (Supplementary Figures 1e, f). Various DNA: PEI ratios were tested, and both 1:3 (Supplementary Figures 1a, b) and 1:5 (Supplementary Figures 1c, d) ratios efficiently transfected the cells. However, the 1:3 DNA: PEI ratio resulted in higher cell viability, which is crucial for maintaining healthy cells during lentiviral production.

**FIGURE 2:**
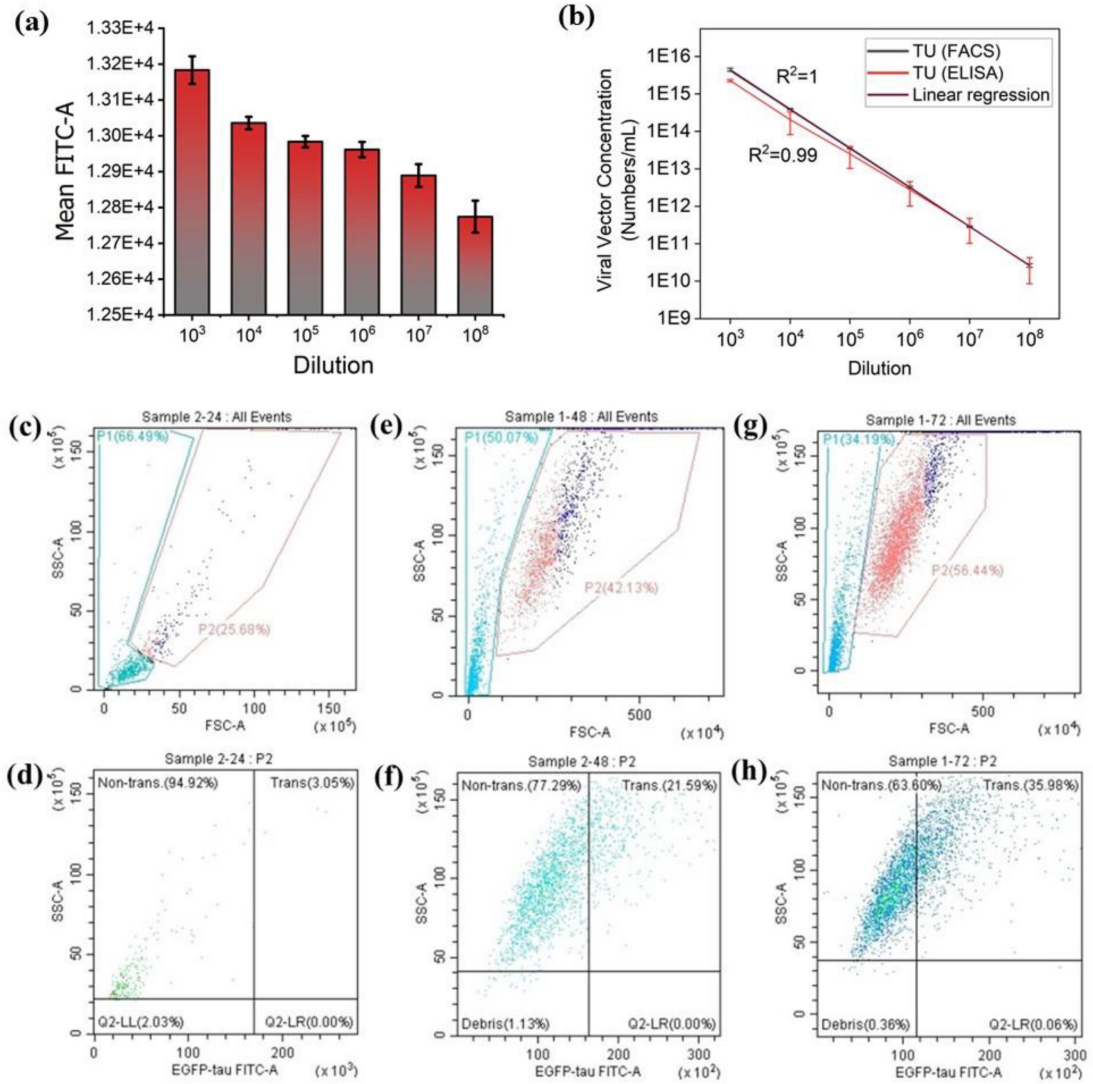
Transduction efficiency in Neuro-2a cells assessed by flow cytometry and ELISA. **(a)** The mean FITC signal across various lentiviral dilutions shows that transduction efficiency decreases with lower viral vector concentrations. **(b)** Titration of concentrated lentiviral vector preparations using ELISA and flow cytometry. Lentiviral vectors were serially diluted and applied to Neuro-2a cells; titration linearity was assessed using both methods post-transduction. **(c–h)** Quantification of EGFP-tau expression in Neuro-2a cells at 24 h **(c,d)**, 48 h **(e,f)**, and 72 h **(g,h)**, following lentiviral transduction. Panels **(c,e,g)** display flow cytometry gating at the respective time points. The highest expression level was observed at 72 h post-transduction. The vertical axis (SSC-A) represents the Side Scatter, while the horizontal axis (EGFP-tau-FITC-A) indicates EGFP-tau fluorescence intensity. “Trans.” shows the EGFP-tau transduced cells, and “non-trans.” indicates non-transduced cells. The threshold for EGFP-tau positivity was set based on the negative control (non-transduced cells).

The flow cytometry results of lentiviral vector titration (Figure 2a) demonstrated a decrease in Mean FITC-A with decreasing viral vector concentration. To determine the optimal lentiviral dilution for subsequent experiments, the viability of transduced cells was assessed using the Trypan Blue Exclusion viability test (Supplementary Figure 2). Notably, a high mean FITC-A is not always ideal, as maintaining low cell toxicity is crucial for optimized experimental conditions. Based on these findings, the 10^6^ dilution was identified as the most suitable lentiviral concentration for transducing Neuro-2a cells, striking a balance between sufficient transduction efficiency and cell viability after exposure to the produced lentiviral vectors.

The lentiviral vectors were quantified using two methods: flow cytometry and p24 antigen ELISA. Some variations in transducing unit measurements between the two methods were observed (Figure 2b), likely due to the transient nature of lentiviral vectors and differences in experimental protocols. While the absolute values may differ, the results should follow the same overall trend (66). Although flow cytometry analysis is dependent on the fluorescent marker and cannot differentiate between single or multiple integrations, it is more reliable than ELISA since it quantifies the viral vectors based on counting the transduced cells and not only P24 which may be either free or on the surface of viral vectors. In other words, the flow cytometry analysis considers only functional vectors while ELISA quantifies both functional and nonfunctional vectors. These results are consistent with the findings reported by Geraerts et al. and Jang et al. (66, 67).

**FIGURE 3:**
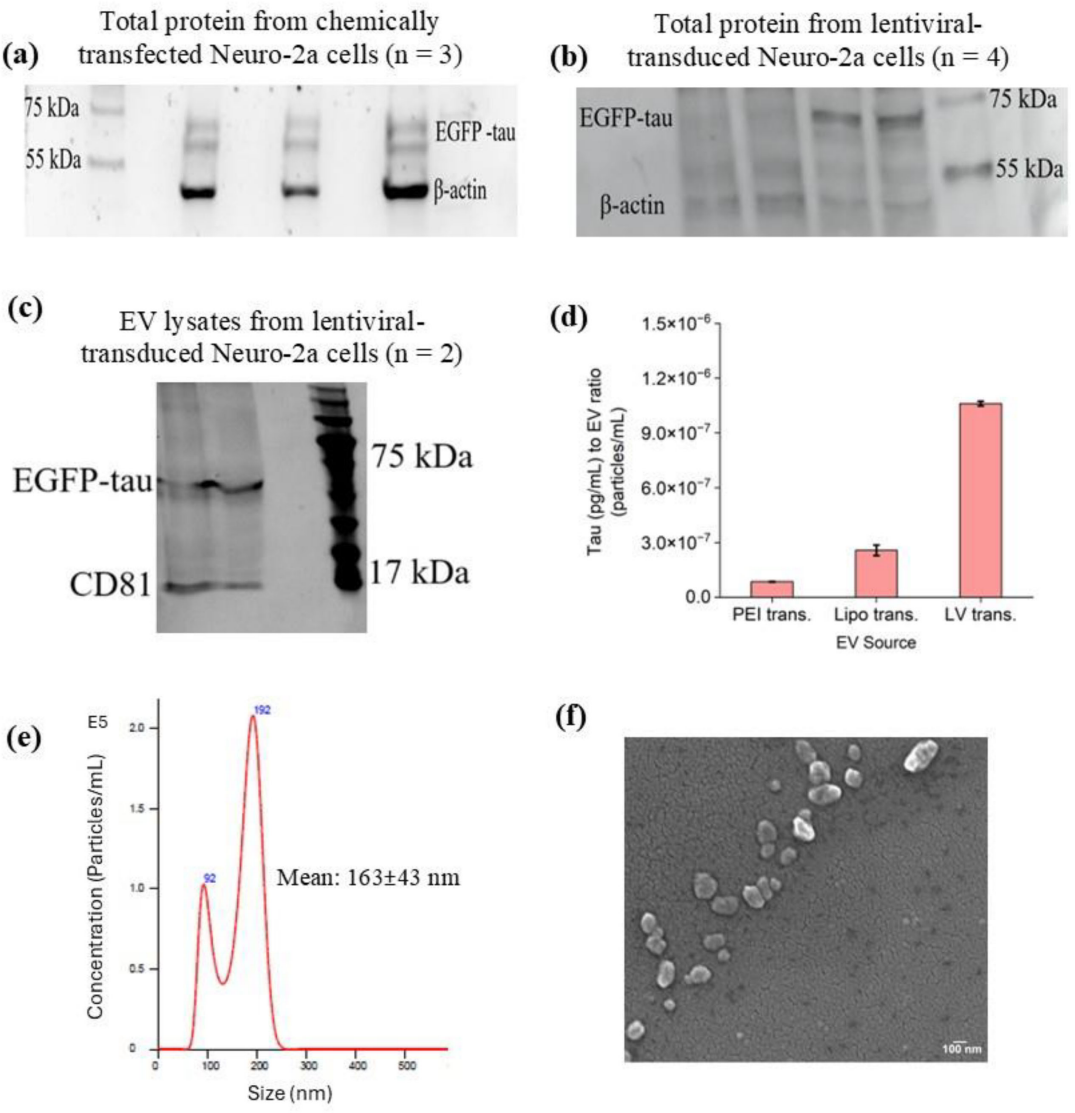
Characterization of tau expression and EV properties following gene delivery. **(a)** Western blot analysis of tau (EGFP-tau) expression in Neuro-2a cells following chemical transfection. **(b)** Western blot analysis of tau expression in Neuro-2a cells after lentiviral transduction. **(c)** Western blot analysis of EVs derived from lentiviral-transduced Neuro-2a cells, confirming the presence of tau protein in EV-associated cargo. β-actin was used as a loading control for all blots. **(d)** Quantification of tau protein levels in EVs isolated from Neuro-2a cells subjected to chemical transfection or lentiviral transduction, as determined by ELISA. Tau concentrations were normalized to EV particle counts (particles/mL). No significant difference was observed in tau loading between the different transfection methods (one-way ANOVA, *p* > 0.05). **(e)** NTA of EVs isolated from Neuro-2a cells, showing a particle size of 163 ± 43 nm and a concentration of 1.25 × 10^8^ particles/mL. **(f)** SEM of EVs, revealing spherical morphology and size consistent with NTA measurements.

To determine the optimal time for tau expression, confluent Neuro-2a cells were transduced with lentiviral vectors at a dilution of 1:1,000,000. Flow cytometry analysis was then performed at various time points, 24 h, 48 h and 72 h, following transduction, to monitor EGFP-tau expression levels (Figures 2c–h). The results indicated that 72 h post-transduction was the optimal time point, as Neuro-2a cells exhibited the highest levels of tau expression at this stage (Figures 2g, h). Next, to confirm the detectable levels of EGFP-tau proteins in our *in vitro* tauopathy models, we analyzed chemically transfected cells (Figure 3a), lentiviral-transduced cells (Figure 3b) and EVs derived from lentiviral-transduced cells (Figure 3c) using Western blotting. The successful transfer of EV lysates proteins to nitrocellulose membranes was validated using both Ponceau staining (Supplementary Figure 3a) and the No-Stain Protein Labeling Reagent (Supplementary Figure 3b). β-actin served as an internal loading control to ensure equal protein loading across samples. To quantify the total extracted protein, a standard curve was generated using a serial dilution of bovine serum albumin as the standard (Supplementary Figure 4). This approach ensured accurate normalization and reliable quantification of tau expression levels.

### 3.2 Tau measurement in EVs derived from different tauopathy models

Measuring tau protein in human samples is challenging, and many growing studies try to develop systems capable of capturing and then measuring different common AD biomarkers. In this study, the tau protein captured in EVs derived from different tauopathy models was measured using a human tau ELISA kit (Figure 3d). Since the EVs yield from different sources, depending on the techniques and cell types, can be different, it is important to consider EV particles/mL for each sample. In this experiment, the tau concentration in each sample (pg/mL) was measured using the prepared standard curve (Supplementary Figure 5) and then normalized to the EV count (particles/mL) to ensure accurate comparison across different conditions. The results of tau loading in EVs under varying transfection methods using ELISA showed no significant difference (one-way ANOVA, *p* > 0.05).

### 3.3 EV characterization

The EVs extracted from transfected Neuro-2a cell samples were characterized using multiple techniques to confirm their size, shape, and quality. NTA revealed that most EVs had an average size of 163 ± 43 nm and a concentration of 12.48 × 10^7^ particles/mL (Figure 3e). DLS analysis indicated a zeta potential of −15.77 mV ± 3.19 mV SD, confirming the stability of the isolated EVs. SEM further validated these findings by revealing EVs as spherical particles with smooth membrane surfaces and an average size consistent with the NTA results, providing visual confirmation of EV morphology and supporting the successful isolation of intact EVs from Neuro-2a cell samples (Figure 3f).

### 3.4 DNA loading efficiency in EV-based tauopathy models

The primary goal of this EV-based gene delivery optimization was to assess the impact of DNA amount (μg) and incubation time (h) on DNA loading efficiency in EVs and gene expression in Neuro-2a cells at both the gene and protein levels. The amount of DNA loaded in EVs after electroporation was measured based on the standard cure prepared (Supplementary Figure 6) using Quant-it PicoGreen assay kit. Notably, since the EV particle number remained constant across all experiments, the DNA loading efficiency results (Supplementary Figure 7) suggest that using a higher amount of plasmid DNA, in the context of the same EV particle number, creates an inappropriate ratio between EV particles and DNA. This imbalance leads to incomplete DNA loading in EVs. Nevertheless, the loaded DNA amount is sufficient to achieve detectable and even high expression levels in Neuro-2a cells. Furthermore, the DNA amount in the transfection mixture after electroporation was normalized to the amount of DNA before electroporation to ensure consistency in the DNA loading process, and these results do not reflect the number of DNA molecules per EV particle. Therefore, the results suggest that EV particles inherently increase their cargo capacity with higher DNA levels and EV particles reorganize their cargo load based on the available DNA amount, with lower DNA levels resulting in fewer DNA molecules per EV particle.

### 3.5 Confocal microscopy validation of tau expression in EV-based tauopathy models

**FIGURE 4:**
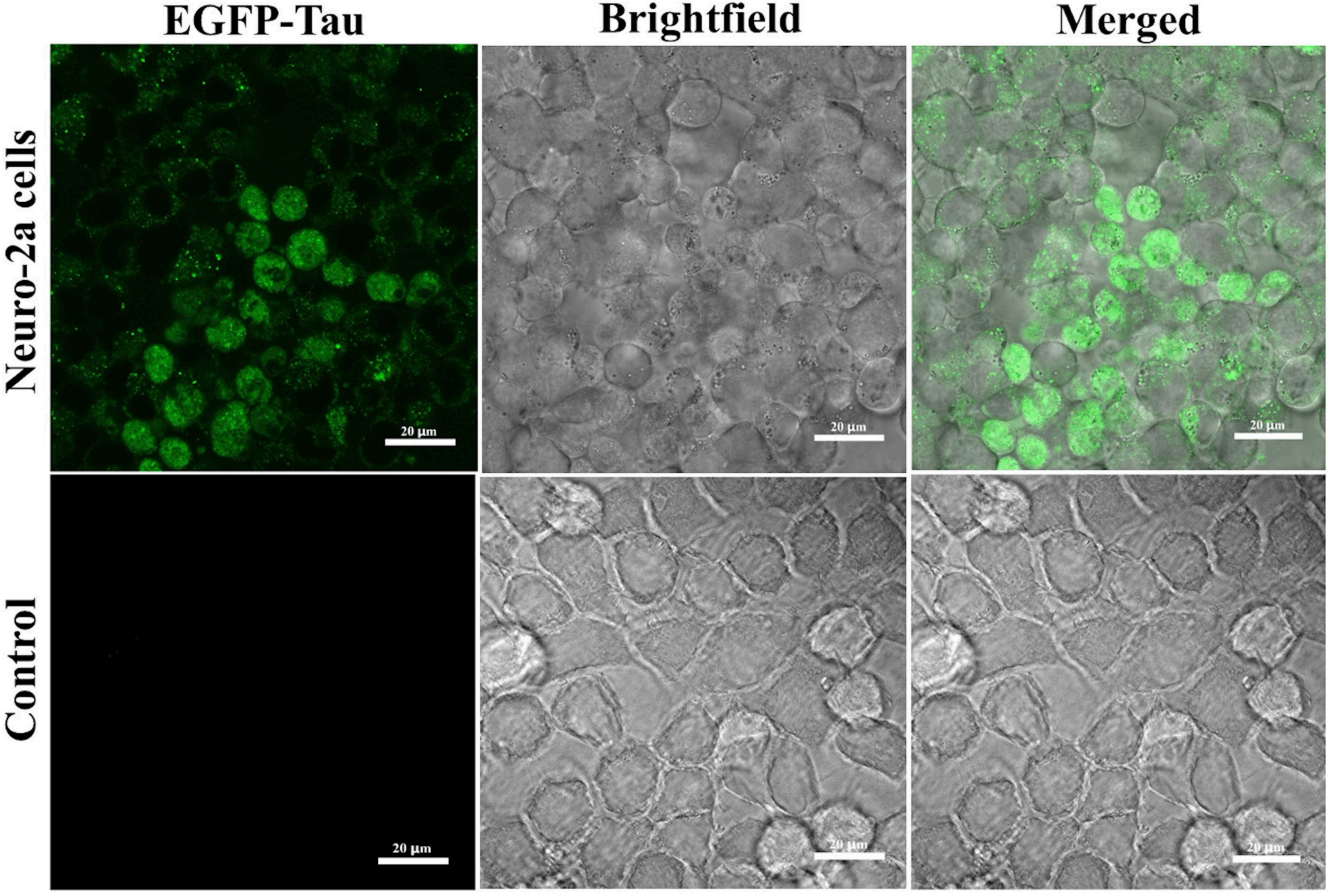
Extracellular vesicle (EV)-mediated EGFP-tau gene delivery in Neuro-2a cells. Confocal microscopy images of Neuro-2a cells treated with EVs encoding EGFP-tau. The EGFP fluorescence signal indicates localization of tau protein within the cells (green). The control treated with non-transducing EVs shows only minimal background fluorescence.

The overexpression of tau was confirmed by imaging transfected Neuro-2a cells using confocal microscopy. The images demonstrated the presence of the fluorescent marker in Neuro-2a cells 48 h after treatment with EV-based transfection mixture, confirming the successful delivery of the EGFP-tau gene via EVs (Figure 4). The EGFP expression levels detected by confocal microscopy highlight the efficiency of EVs in successfully localizing the tau protein in Neuro-2a cells.

### 3.6 Quantification of tau production

To validate the confocal microscopy results and further quantify the fluorescence intensity of the EGFP reporter, flow cytometry analysis was performed. The normalized median FITC fluorescence intensity for each experiment is presented in Supplementary Figure 8a, alongside the high cell viability percentages observed in all experiments (Supplementary Figure 8b). These results indicate that when the DNA amount exceeded 10 μg, the median FITC response was extremely high. Additionally, this response further increased with longer incubation times (h) in experiments one and two using the same DNA amount. These results confirm the synergistic effect of both DNA amount (μg) and incubation time (h) on the successful delivery and expression of tau in Neuro-2a cells using the EV-based transfection system. No cell toxicity was observed 48 h after treating Neuro-2a cells with up to 15 μg of plasmid DNA. Additionally, even 24 h of exposure to the transfection mixture did not affect cell viability, demonstrating the biocompatibility of the EV-based transfection mixture, which contains no chemical transfection reagents.

### 3.7 Quantification of tau mRNA using qRT-pCR

To assess the impact of DNA amount (μg) and incubation time (h) on tau expression, qRT-PCR was performed to quantify tau gene expression levels relative to GAPDH. The fold change results (Supplementary Figure 9), calculated using 2^∧^(−ΔΔCt), revealed notable variations in experiments with more than 8 μg of DNA, such as experiments 1, 2, and 10. Consistent with the flow cytometry results, these findings indicate that increasing the incubation time (h) significantly enhances mRNA levels in EV-based transfected Neuro-2a cells.

### 3.8 Assessment of data normality, model fit, and statistical analysis

**FIGURE 5:**
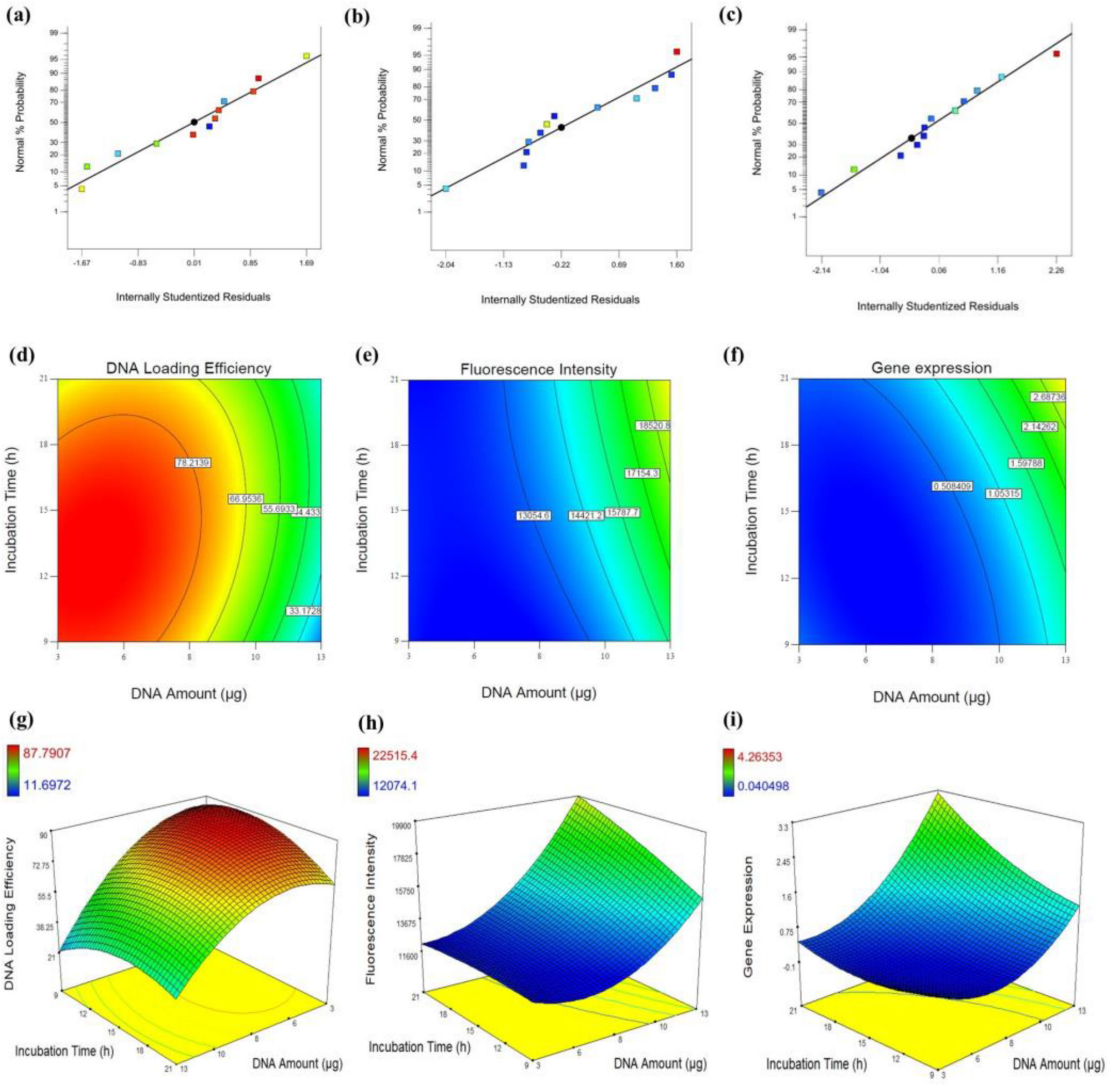
Assessment of DNA loading efficiency, fluorescence intensity, and gene expression in Neuro-2a cells. **(a–c)** Normal probability plots of internally studentized residuals for DNA loading efficiency **(a)**, fluorescence intensity **(b)**, and gene expression **(c)**, indicating the adequacy of the regression models. **(d–f)** Contour plots illustrating the interactive effects of DNA amount (μg) and incubation time (h) on DNA loading efficiency **(d)**, fluorescence intensity **(e)**, and gene expression **(f)**. **(g–i)** Corresponding 3D response surface plots depicting the same interactions for DNA loading efficiency **(g)**, fluorescence intensity **(h)**, and gene expression **(i)**.

To ensure a normal distribution of error terms, a normal probability plot was generated, showing a straight line that helps confirming the normality of responses, including DNA loading efficiency, fluorescence intensity, and gene expression level. As shown in the plots in Figures 5a–c, most points align closely with a straight line, indicating that the data follows an approximately normal distribution in all three responses. The high R^2^ values of 0.9409 for plasmid DNA amount, 0.9201 for fluorescence intensity, and 0.8028 for gene expression level, suggest that the modified quadratic models provide a reliable fit for the experimental system. According to Joglekar and May (68), an R^2^ value greater than 0.80 is considered acceptable for a model to be considered a good fit.

The Equations 1–3 define the models predicted by the coded factors [DNA amount (μg) and incubation time (h)] for each response: DNA loading efficiency, fluorescence intensity, and gene expression level:

Response 1 (R1): DNA loading efficiency:


$$R\,1=80.45-23.89\,A-1.72\,B+7.59\,A\,B-18.12\,A^{2}-10.66\,B^{2}$$ (1)

Response 2 (R2): Fluorescence intensity:


$$\begin{array}{cc} R\,2 & =13250.05+2834.08\,A+947.27\,B+1053.36\,A\,B+\\ & 2026.37\,A^{2}-223.73\,B^{2} \end{array}$$ (2)

Response 3 (R3): Gene expression level:


$$R\,3=0.26+0.91\,A+0.47\,B+0.49\,A\,B+0.80\,A^{2}+0.30\,B^{2}$$ (3)

In Equations 1–3, A and B represent DNA amount (μg) and incubation time (h), respectively. All equations demonstrate that DNA amount is a more significant factor than incubation time both in fluorescence intensity and gene expression levels. However, the interaction between DNA amount (μg) and incubation time (h) is more influential in fluorescence intensity. Notably, the high significance of the incubation time reflects the importance of providing cells with sufficient exposure to the transfection mixture, allowing enough time for EVs to traverse the cell membrane and facilitate the integration of transferred DNA in the cellular expression processes. It shouldn’t be neglected that the non-linear effect of DNA amount (A^2^) has a strong negative effect on DNA loading efficiency but strong positive effects on fluorescence intensity and gene expression. In addition, the negative impact of DNA amount on DNA loading efficiency contrasts with its positive effects on fluorescence intensity and gene expression, suggesting a potentially meaningful tradeoff in the system’s performance. Such tradeoffs are common and acceptable, particularly in systems where multiple responses are being optimized simultaneously. In this case, the observed tradeoff may be attributed to an imbalanced DNA: EV particle ratio in experiments with higher DNA levels. Despite this imbalance, the amount of loaded DNA remains sufficient to achieve detectable gene expression in host cells. In systems like this, improving one outcome can detrimentally affect another due to the system’s inherent constraints or dynamics. The interactive effects of DNA amount (μg) and incubation time (h) on each response are visually represented in the contour and 3D graphical maps shown in Figures 5d–i, providing valuable insights into the interplay between these factors. The 3D curve in Figure 5g depicts the interaction between DNA amount (μg) and incubation time (h), and their influence on DNA loading efficiency. The data shows that higher DNA amount (higher than 8 μg), regardless of incubation time (h) was not suitable for DNA loading efficiency. This may be due to an inappropriate balance between the amount of DNA and the number of EV particles. This suggests the existence of an optimal DNA-to-EV ratio for efficient loading.

Additionally, extending incubation time (h) does not significantly enhance DNA loading into EVs, even after electroporation. In contrast, both contour plots for fluorescence intensity and gene expression levels (Figures 5h, i) demonstrate a strong correlation between these two parameters. As the DNA amount (μg) increases, both responses show an upward trend, which is further amplified by prolonged incubation time (h). Overall, these findings suggest that, despite the low DNA loading efficiency in EVs, the amount of DNA transferred per EV particle was sufficient to induce detectable tau expression in Neuro-2a cells.

To evaluate the variances in the models proposed by the Response Surface Methodology (RSM), a quadratic Analysis of Variance (ANOVA) was conducted for each response (Supplementary Table 3). For DNA loading efficiency, the Model *F*-value of 19.09 and an Adeq Precision ratio of 12.081 indicate a statistically significant and robust model with significant terms A, A^2^, and B^2^. The “Lack of Fit *F*-value” of 1.01 suggests that the lack of fit is not significant, with a 49.64% probability of such a value arising due to noise, confirming the model’s accuracy. For fluorescence intensity, the Model *F*-value of 13.82 and an Adeq Precision ratio of 11.311 demonstrate a reliable model with significant terms A and A^2^. The “Lack of Fit *F*-value” of 3.11 further supports that the model fits well, with an 18.84% probability of the lack of fit occurring due to noise. Finally, for gene expression, the Model *F*-value of 4.89 indicates statistical significance with A and A^2^ as key contributors. The “Lack of Fit” is not significant, which is desired. However, it has a lower probability (5.34%) compared to other responses which might be due to some effective variations such as EV concentration which is a fixed parameter in this model but might be considered for future gene delivery-based models. To determine the optimal conditions, an optimization scenario was tested in which all three responses were targeted to retain the maximum goals with high importance. The model suggested optimized conditions of 11.34 μg for plasmid DNA and 19.56 h for incubating the cells with the transfection mixture, resulting in a DNA loading efficiency of 53%, fluorescence intensity of 17157.8 and a qRT-PCR fold change of 1.97. Interestingly, even when DNA loading efficiency was considered a low-impact factor, an efficiency of 53% can result in strong gene expression in Neuro-2a cells. This suggests that under conditions with lower particle numbers but higher DNA content per EV particle, each EV particle likely carries more DNA molecules.

To validate the model’s prediction, an experiment was conducted under the optimized conditions mentioned above. The experimental results showed the DNA loading efficiency of 69.74%, the fluorescence intensity of 17059.6 and a qRT-PCR for fold change of 2 show that they align well with RSM’s predicted values, indicating that the model can accurately predict the experimental responses. In summary, creating effective tauopathy models for AD involves a biocompatible gene delivery system for tau. EVs offer innate capabilities for intercellular DNA transport, making them highly promising for gene therapy. This study optimized EV-mediated delivery of tau plasmid DNA into Neuro-2a cells. Results indicate that EVs effectively deliver large plasmid DNA, achieving significant gene expression and emphasizing their potential as a reliable alternative to conventional techniques.

## 4 Discussion

This study demonstrates that EVs can be used as efficient carriers for the tau gene in Neuro-2a cells, achieving detectable tau protein expression. Our optimization using RSM highlights how varying plasmid DNA amount (μg) and incubation time (h) impacts DNA loading efficiency, fluorescence intensity, and qRT-PCR fold change in EV-based transfection system. Specifically, our results show that even with moderate DNA loading efficiency, significant gene expression was achieved, suggesting a high DNA-to-EV ratio per particle. This finding aligns with Kang et al. (46), who found that DNA loading in EVs is contingent on DNA size and dose, emphasizing EVs’ adaptability as gene carriers.

Our findings also complement the work of Lamichhane et al. (47), who observed size-dependent DNA loading efficiency in EVs, with smaller EVs more effectively associating with DNA fragments under 1000 base pairs, while larger EVs could carry plasmids. In this study, successful gene expression was observed despite the large size of the tau plasmid, suggesting that EVs can indeed support the delivery of sizable DNA constructs for effective transgene expression. This reinforces the viability of EVs as carriers for larger genetic material, extending the conclusions of Kahlert et al. (43), who documented the capacity of EVs to hold double-stranded DNA beyond 10 kb.

Although several delivery studies have focused on proteins and microRNAs (69–71), the presence of double-stranded DNA in EVs, both membrane-associated DNA (larger than 2.5 kb) and encapsulated DNA (100 bp–2.5 kb) demonstrates their ability to transport large DNA (45). Tsering et al. (72) reviewed the strong potential of EVs in gene therapy and vaccine applications, although clinical use of EV-mediated DNA delivery faces challenges such as cargo heterogeneity, loading efficiency, and transient expression of delivered DNA (72). For example, successful EV-mediated delivery of p53 *in vitro* (p53-null H1299 cells) and *in vivo* (p53-knockout mice) restored p53 expression in recipient cells (73). Bone marrow mesenchymal stem cell–derived EVs, either alone or hybridized with liposomes, successfully transferred large plasmid DNA, such as the Cas9-green fluorescent protein plasmid (74). Delivery of Herpes simplex virus thymidine kinase plasmid DNA by EV-based hybrid systems into the brain for glioblastoma therapy provides another example of EVs transporting large plasmids (75). Given the variability in the field, standardization, transparency, and data sharing remain essential (72). Our optimization study can improve our understanding of EV behavior in DNA delivery and guide the initial steps in optimizing gene therapy.

A key advantage of EVs lies in their ability to overcome barriers typical of other transfection methods, as shown in various studies. For instance, Ortega-Pineda et al. (50) and Rincon-Benavides et al. (51) demonstrated the efficacy of EVs in reprogramming cells, such as fibroblasts to neurons, using bulk electroporation to load DNA plasmids in EVs. These studies confirm that EVs not only deliver genetic material but also support complex cell fate changes, suggesting potential applications for EV-mediated gene delivery in AD research, where tau pathology modeling requires stable expression of tau-related genes in neuronal cells. Compared to chemical methods like Lipofectamine, which has been used in the human neuroblastoma cells for similar purposes but carries risks of cytotoxicity (14, 76), EVs offer a less toxic alternative with comparable or superior transfection efficiency. While viral vectors, especially lentivirus, are effective for long-term gene expression (14), they come with risks of insertional mutagenesis and immune responses. Our results indicate that EVs can achieve a level of gene expression that is both effective and safer. However, it should be noted that a limitation of this study is the absence of a direct, quantitative comparison between EV- and lentiviral-mediated gene delivery under identical conditions. Future studies should address this gap to enable more robust conclusions regarding relative efficiency and safety.

In the context of AD, the successful delivery of tau through EVs supports previous studies, like that of Alvarez-Erviti et al. (48), who achieved targeted delivery of therapeutic siRNA to the brain in AD models using EVs. Our approach provides a similar platform for studying tau pathology with the advantage of low immunogenicity and an ability to cross biological barriers, making it suitable for neurological applications. Furthermore, EVs’ potential to deliver gene-editing tools opens new avenues for precise manipulation of gene expression in tauopathy models, which could be invaluable in unraveling the molecular mechanisms of tau aggregation and AD progression.

Our findings contribute to a growing body of evidence supporting EVs as effective, biocompatible gene delivery systems. Future work should further explore the scalability of EV-based transfection methods to enhance DNA carrying capacity and expression efficiency. Collectively, this study provides new insights into the use of EVs for gene delivery, offering promising directions for developing more effective tauopathy models and exploring gene therapy approaches for AD and other neurodegenerative diseases.

## 5 Conclusion

The findings from this study demonstrate that variations across the 12 experimental conditions yielded statistically significant differences in fold change, EV loading, and fluorescence intensity, underscoring the impact of two variants including the DNA amount (μg) and the incubation time (h) on tau gene delivery by the EVs extracted from the Neuro-2a cells. These results reveal a potential mechanism by which EV-based delivery influences the cellular gene expression level, providing novel insights that may inform future strategies in gene delivery at both scientific and clinical levels for diagnostic and therapeutic purposes. While the diversity of conditions in this study offered a strong basis for initial conclusions, additional research is recommended to validate these findings. Overall, this research contributes to a deeper understanding of safe and quick gene delivery, offering fundamental applications such as personalized delivery using EVs from the host cells, not only for gene delivery but also for drug delivery.

## Data Availability

The raw data supporting the conclusions of this article will be made available by the authors, without undue reservation.
