# Supplementary material for: Developing and optimizing a biocompatible tauopathy model using extracellular vesicle-mediated gene delivery
Source: Front Med (Lausanne). 2025 Oct 22;12:1672046. doi: 10.3389/fmed.2025.1672046 (PMC12587183; doi:10.3389/fmed.2025.1672046)
Supplement: Supplementary file 1 [file Supplementary_file_1.docx]

**Developing and optimizing a biocompatible tauopathy model using extracellular vesicle-mediated gene delivery**

*Samaneh Ghadami^1^, Kristen Dellinger^1, *^*

^1^Department of Nanoengineering, Joint School of Nanoscience and Nanoengineering, North Carolina A&T State University, Greensboro, NC, United States

*CORRESPONDENCE: Kristen Dellinger, kdellinger@ncat.edu


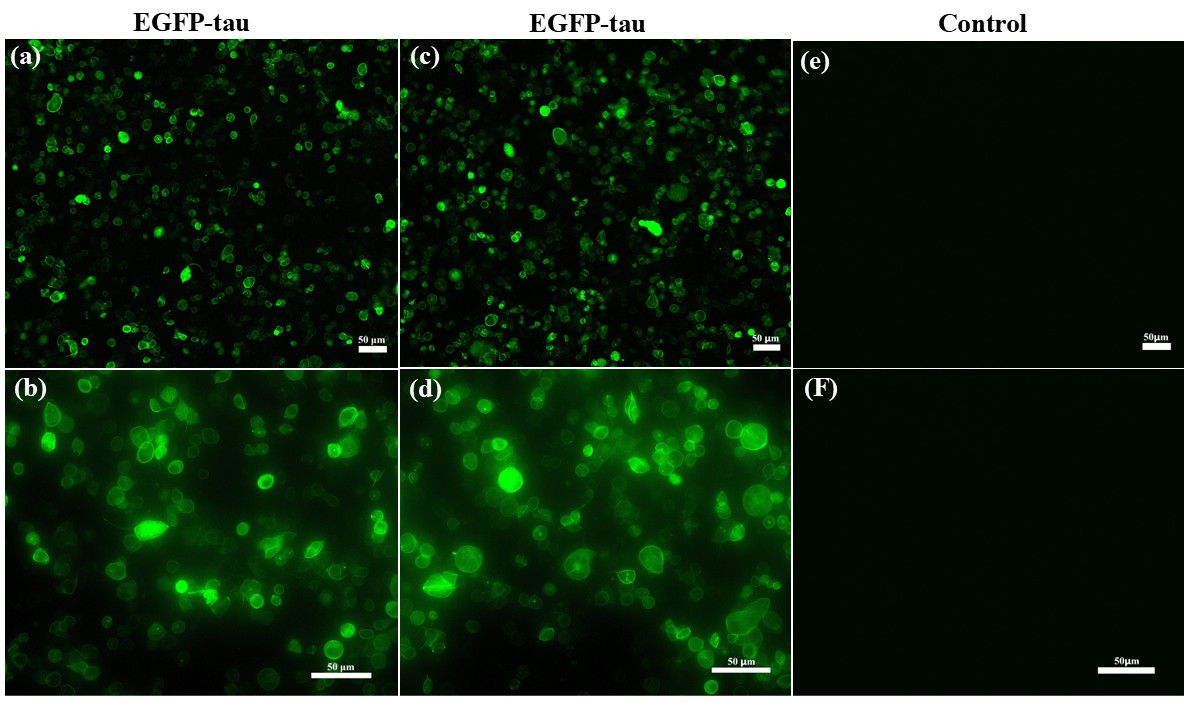


**Figure S1. Tau expression in HEK (p7) cells, 48 hours post-transfection, observed using the Discovery Echo rotary fluorescent microscope.** (a, b) DNA:PEI ratio of 1:3. (c, d) DNA:PEI ratio of 1:5. Both DNA:PEI ratios resulted in effective transfection, with the 1:3 ratio showing higher cell viability compared to the 1:5 ratio. (e, f) Control samples show no EGFP fluorescence signal for both 1:3 and 1:5 DNA:PEI ratios when DNA was excluded from the transfection mix.


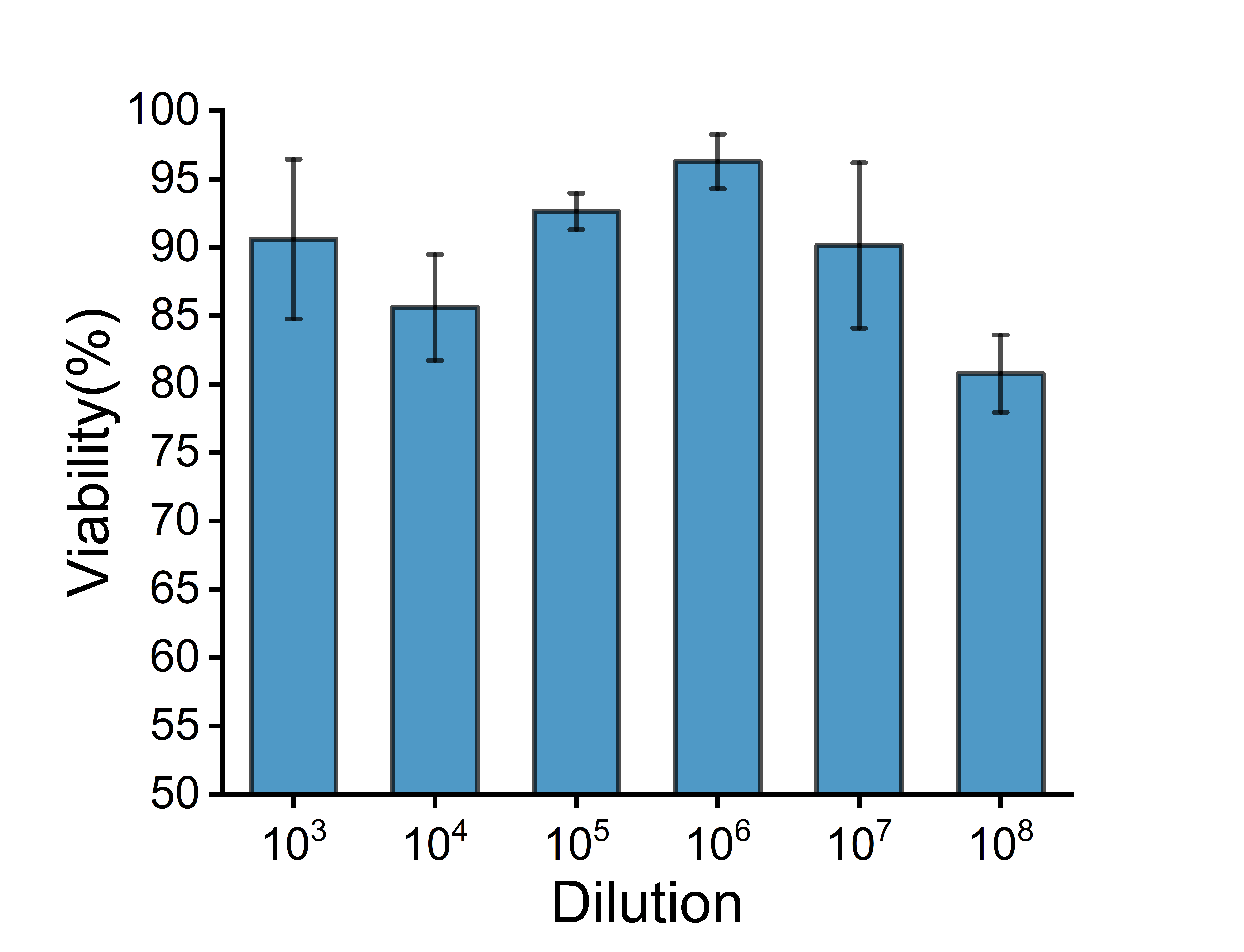


**Figure S2.** **Cell viability was assessed using the Trypan Blue Exclusion Test**. Cell viability was measured 72 hours after exposure to a transfection mixture containing different dilutions of lentiviral vectors. The graph shows that the 10⁶ dilution resulted in the lowest toxicity during transduction of Neuro-2a cells in a 6-well plate.


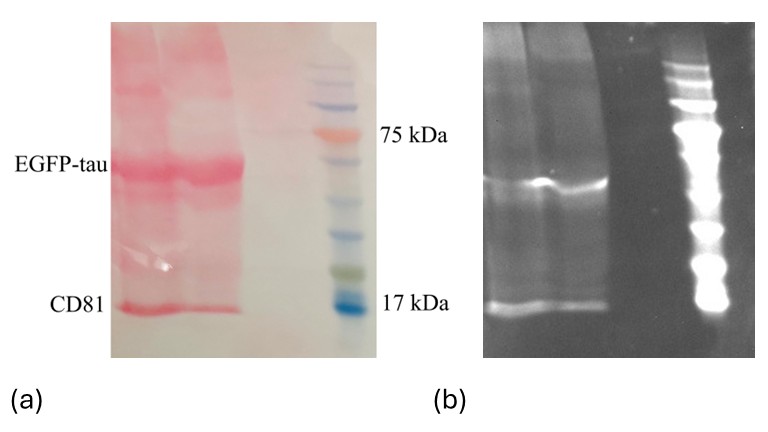


**Figure S3. Verification of protein in EV lysates by Western blotting (n = 2).** (a) Ponceau S staining; (b) No-Stain Protein Labeling Reagent


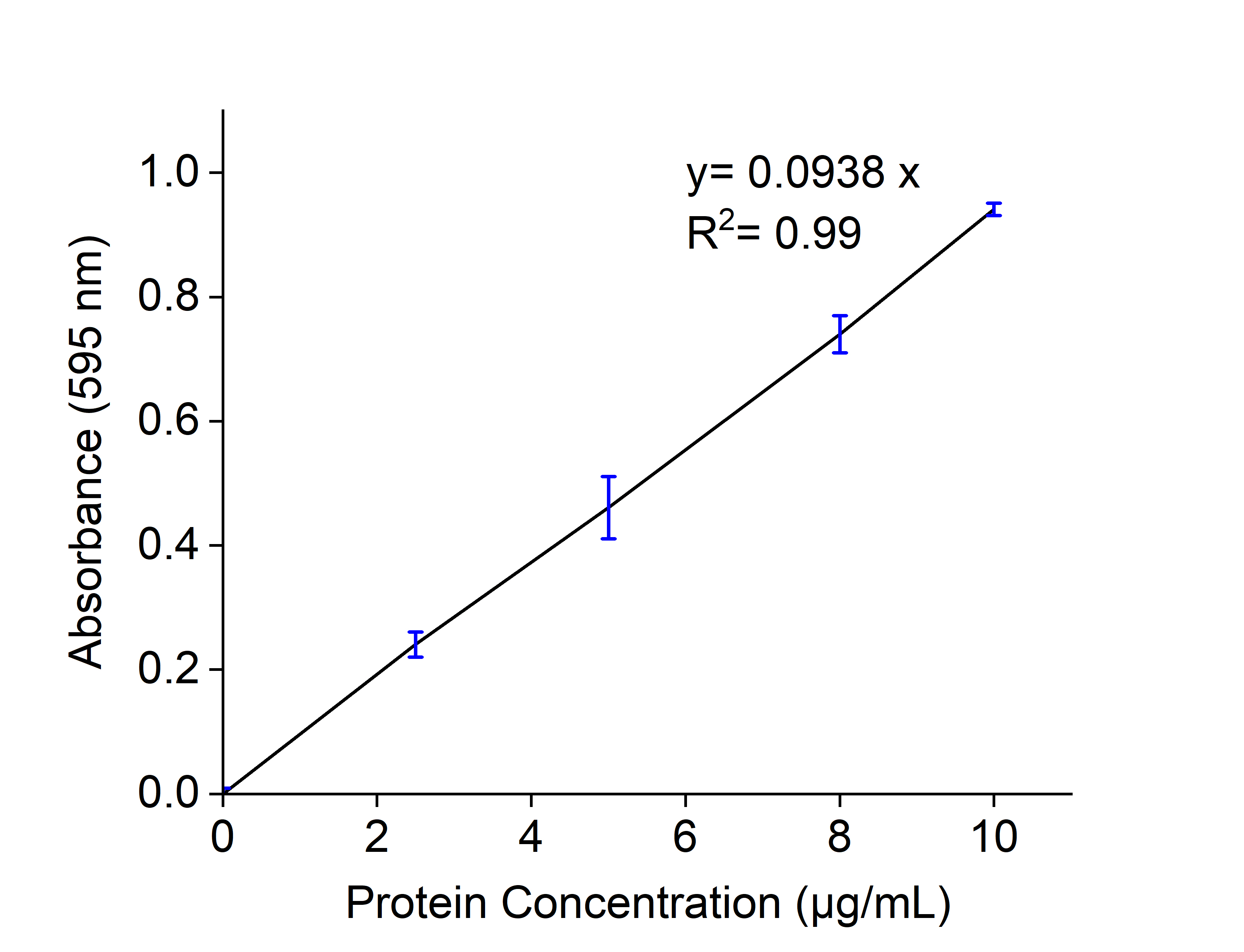


**Figure S4.** Standard colorimetric response curves for bovine serum albumin (BSA) generated using Pierce Bradford Plus Protein Assay Kit, used to determine protein concentrations for Western blotting.


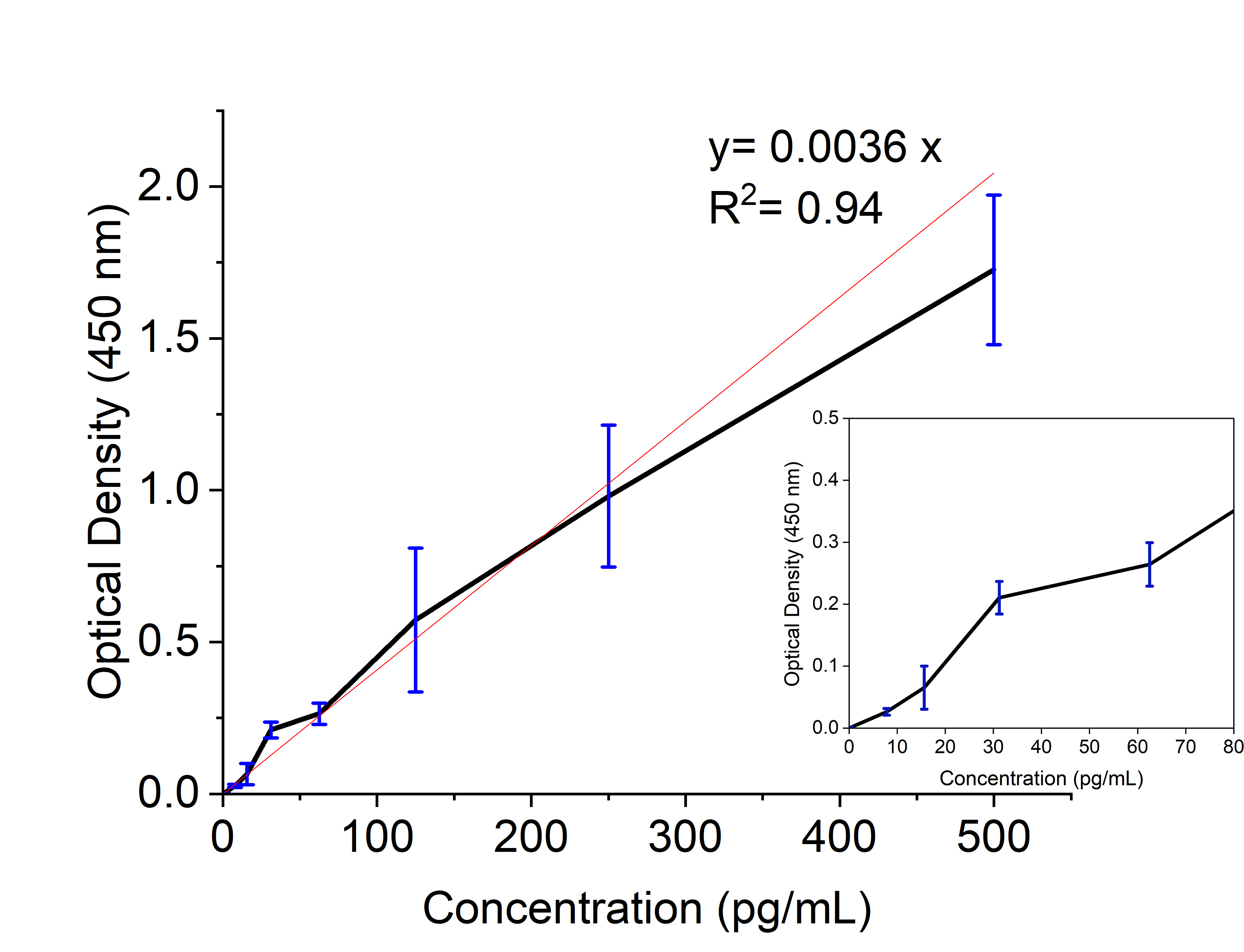


**Figure S5.** The colorimetric response curve for human tau concentration (the range: 7.813-500 pg/mL) using the Novus Biologicals human tau ELISA kit.


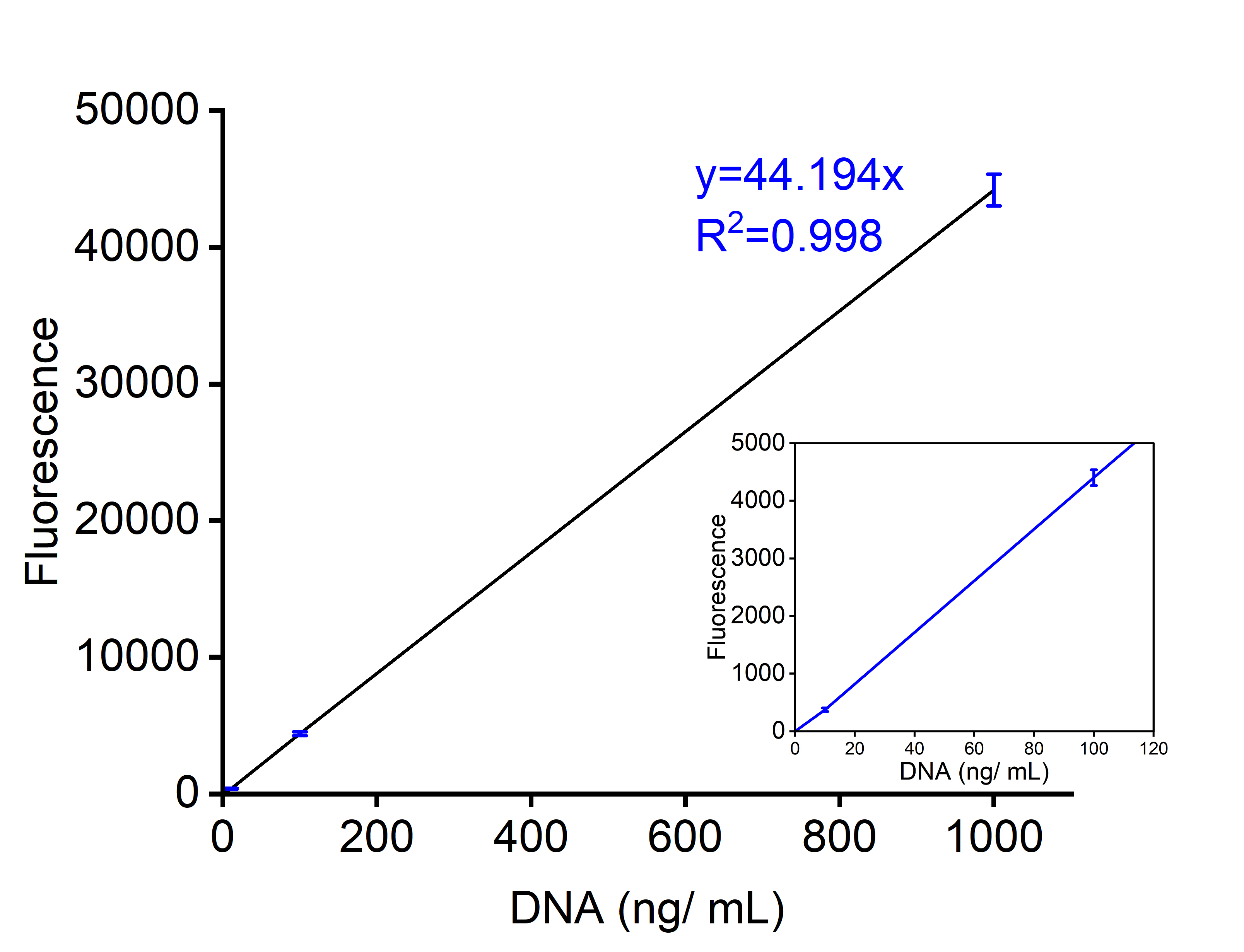


**Figure S6.** High-range standard curve (10 ng/mL to 1 μg/mL) plotted using various concentrations of lambda DNA standard to quantify DNA content in EVs using Quant-it PicoGreen assay kit.


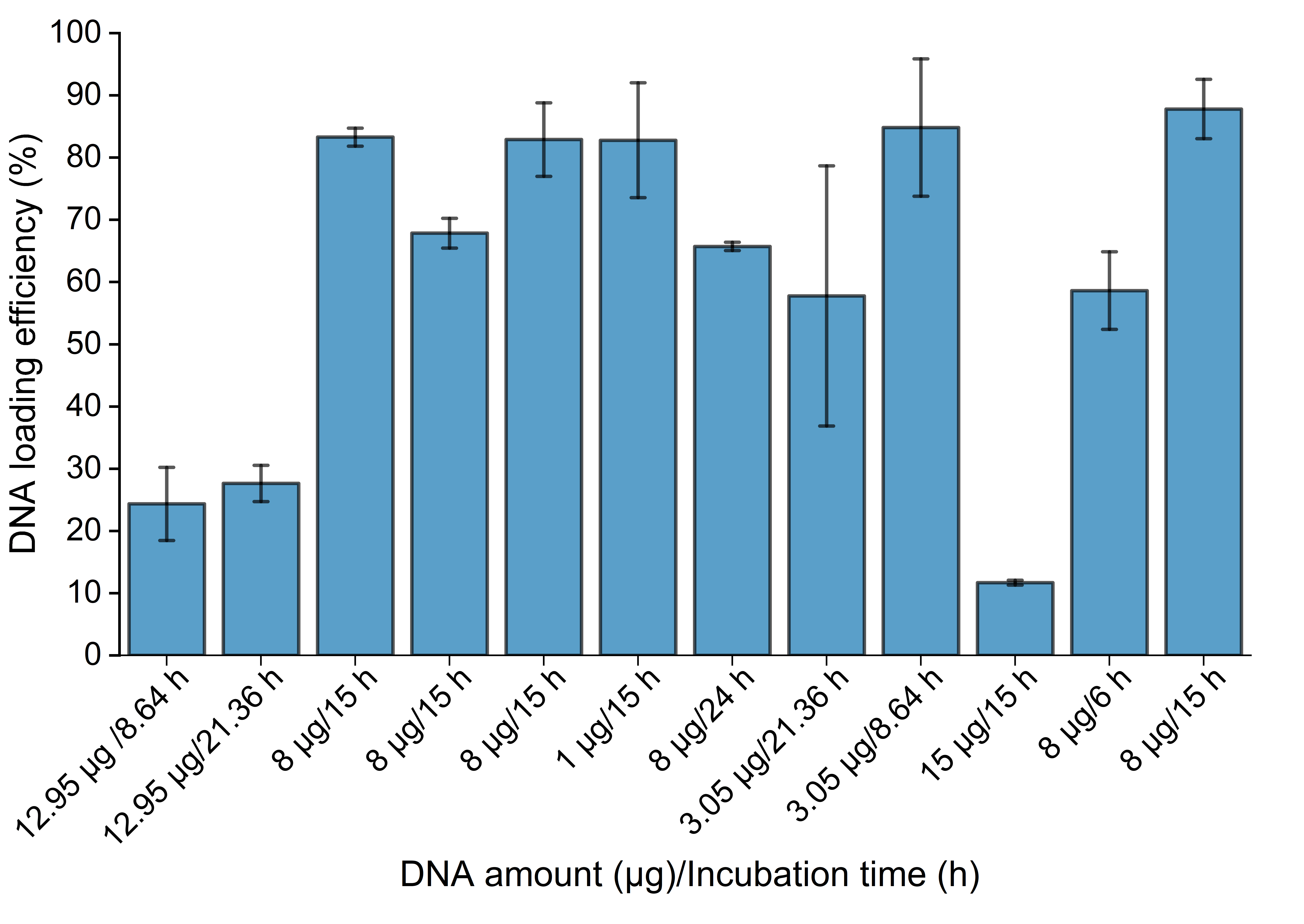


**Figure S7.** **DNA loading efficiency in EVs with varying plasmid DNA amounts (μg) using Quant-it PicoGreen assay kit.** Despite a constant EV particle number, higher DNA amounts led to an imbalance in the EV-to-DNA ratio, resulting in incomplete loading per EV particle. However, sufficient DNA was loaded to achieve detectable expression in Neuro-2a cells.


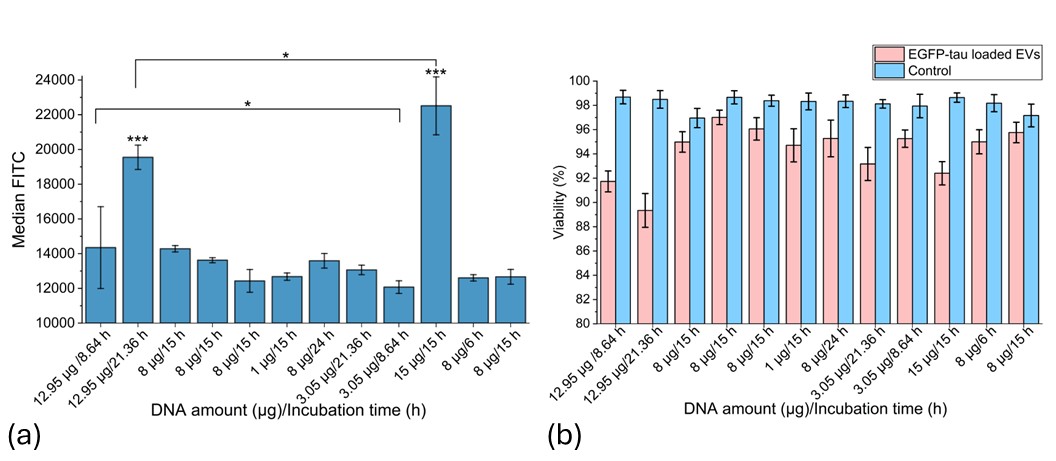


**Figure S8. Fluorescent Intensity and Viability in EV-Based Gene Delivery**

(a) Median FITC fluorescence intensity comparison across experiments with varying incubation times and tau DNA amounts. (b) Neuro-2a cell viability, assessed using Trypan blue exclusion, 48 hours post-exposure to the transfection mix. Blue bars represent controls exposed to unloaded EVs, while pink bars indicate cell viability after exposure to tau-loaded EVs. Cell viability varies with changes in DNA amount and incubation time. Data are expressed as mean ± SD (n = 3).


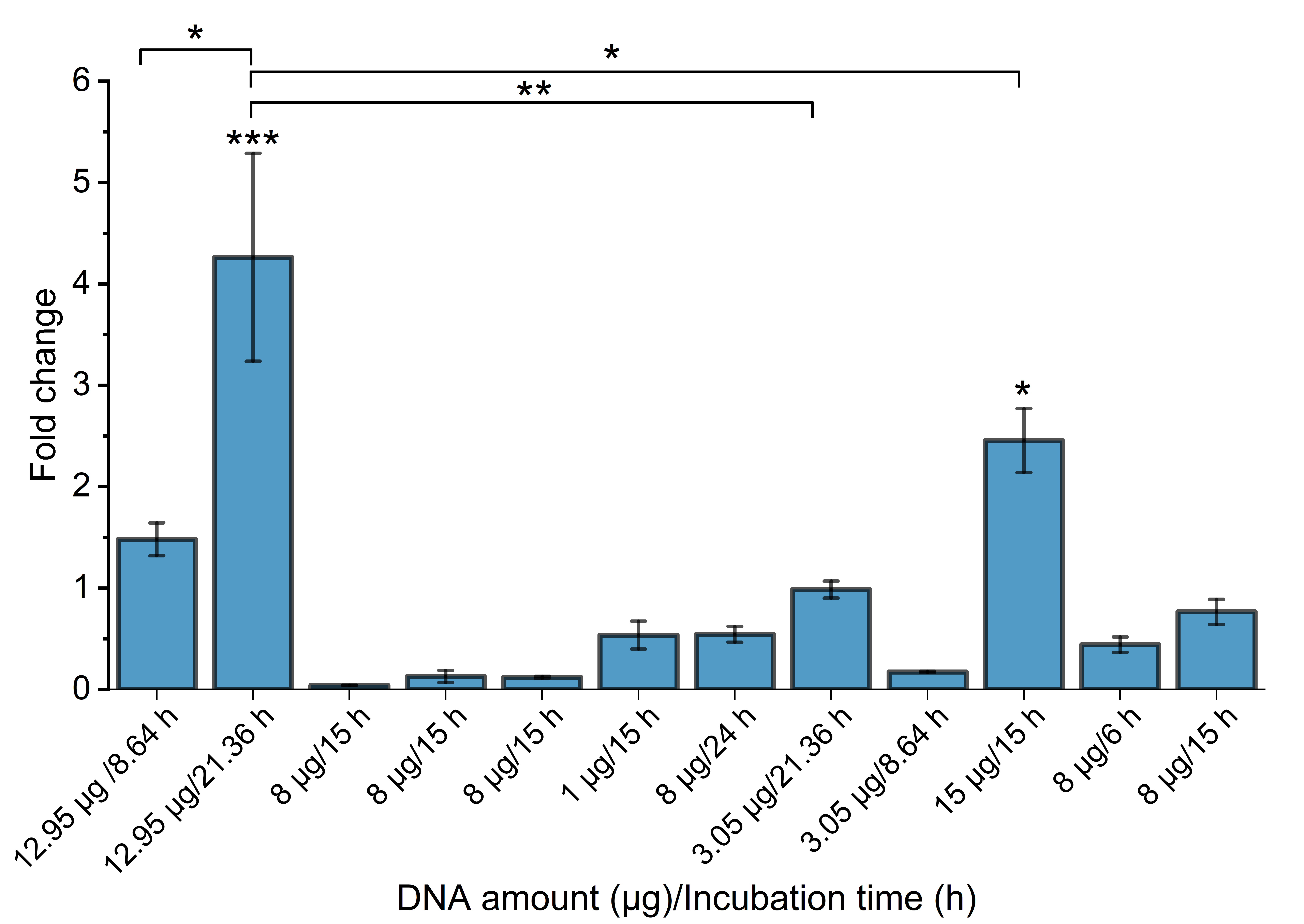


**Figure S9. Relative expression of tau mRNA normalized to GAPDH, measured by qRT-PCR and calculated using the 2^(-∆∆Ct) method.** The graph shows fold changes in tau expression across different DNA amounts and incubation times. Notably, experiments with DNA concentrations greater than 8 μg (Experiments 1, 2, and 10) exhibit fold changes of ≥1, indicating increased tau gene expression at higher DNA levels. Data are expressed as mean ± SD (n = 3).

**Table S1. The experiment details were designed by Central Composite Design (CCD) to propose a model for EV-based gene delivery in Neuro-2a cells.** The experiments with different DNA concentrations and incubation times were designed using CCD for three responses, including tau loading efficiency, fluorescence intensity, and gene expression.

| Runs | Plasmid DNA amount (μg) | Incubation Time (Hour) |
| --- | --- | --- |
| 1 | 12.95 | 8.64 |
| 2 | 12.95 | 21.36 |
| 3 | 8.00 | 15.00 |
| 4 | 8.00 | 15.00 |
| 5 | 8.00 | 15.00 |
| 6 | 1.00 | 15.00 |
| 7 | 8.00 | 24.00 |
| 8 | 3.05 | 21.36 |
| 9 | 3.05 | 8.64 |
| 10 | 15.00 | 15.00 |
| 11 | 8.00 | 6.00 |
| 12 | 8.00 | 15.00 |

**Table S2.** Experimental setup detailing the variation of plasmid DNA amount and incubation time across five levels (-α, -1, 0, +1, +α).

| Variable | Unit | Symbol | Low axial  (-α) | Low factorial  (-1) | Center point  (0) | High factorial  (+1) | High axial  (+α) |
| --- | --- | --- | --- | --- | --- | --- | --- |
| Plasmid DNA | μg | A | 1 | 3.05 | 8 | 12.95 | 15 |
| Incubation Time | h | B | 6 | 8.64 | 15 | 21.36 | 24 |

**Table S3.** ANOVA Analysis of the RSM Model for Optimizing tau Delivery in Neuro-2a Cells Using EVs. This analysis evaluates the two-way interaction between tau concentration and incubation time, examining their effects on three responses: DNA loading efficiency, fluorescence intensity, and gene expression. The results confirm the accuracy and significance of the proposed model for gene delivery optimization. *: The star shows the factor in that response is significant.

| Responses | DNA loading efficiency | | Fluorescence intensity | | Gene expression | |
| --- | --- | --- | --- | --- | --- | --- |
| Variance factors | **F value** | **p-value** | **F value** | **p-value** | **F value** | **p-value** |
| Model | 19.09 | 0.0013* | 13.82 | 0.0030* | 4.89 | 0.0396* |
| A: Plasmid DNA Amount | 60.12 | 0.0002* | 42.38 | 0.0006* | 11.97 | 0.0135* |
| B: Incubation Time | 0.31 | 0.5970 | 4.73 | 0.0725 | 3.14 | 0.1268 |
| AB | 3.03 | 0.1322 | 2.93 | 0.1379 | 1.74 | 0.2351 |
| A^2 | 27.67 | 0.0019* | 17.33 | 0.0059* | 7.34 | 0.0351* |
| B^2 | 9.57 | 0.0213* | 0.21 | 0.6619 | 1.02 | 0.3515 |
| Lack of fit | 1.01 | 0.4964 | 3.11 | 0.1884 | 8.82 | 0.0534 |
| R square | 0.9409 | | 0.9201 | | 0.8028 | |
